# Workplace Exposures May Mask Wildfire Smoke-Related Exposure Inequities and Mortality

**DOI:** 10.1021/acs.estlett.6c00273

**Published:** 2026-06-30

**Authors:** Abas Shkembi, Sara D. Adar, Richard L. Neitzel, Marissa L. Childs

**Affiliations:** † Department of Environmental Health Sciences, 51329 University of Michigan School of Public Health, 1415 Washington Heights, Ann Arbor, Michigan 48109, United States; ‡ Department of Epidemiology, University of Michigan, 1415 Washington Heights, Ann Arbor, Michigan 48109, United States; § Center for Occupational Safety and Health Engineering,University of Michigan, Ann Arbor, Michigan 48109, United States; ∥ Department of Environmental and Occupational Health Sciences, University of Washington School of Public Health, 3980 15th Ave NE, Seattle, Washington 98195, United States

**Keywords:** environmental justice, occupational health equity, social determinants of health

## Abstract

Millions of outdoor workers cannot avoid wildfire smoke, likely leading to inequalities in exposure and health risk, but we lack a comprehensive understanding of how many outdoor workers are exposed to wildfire smoke in the United States (US). We first characterized work-related exposure to wildfire PM_2.5_ for 3,108 contiguous US counties during 2006–2019 by integrating data on wildfire smoke-specific PM_2.5_, workplace activities, and employment counts. We then compared the racial and ethnic composition of each county with the person-days exposed to ambient and work-related wildfire smoke to investigate whether certain racial and ethnic groups experience differential exposure to wildfire smoke at home and at work. Lastly, we replicated a previous analysis of ambient wildfire smoke impacts on all-cause mortality and stratified it by work-related exposure to understand differential mortality effects of ambient wildfire PM_2.5_. Despite experiencing less ambient exposure to wildfire PM_2.5_, counties with higher portions of non-Hispanic Black and Hispanic Americans experienced higher work-related exposures. We also find suggestive evidence that the effect of ambient smoke fine particulate matter (PM_2.5_) concentrations on all-cause mortality may differ by workplace exposure. These findings suggest that workplace exposures should be considered in wildfire smoke-adaptation measures.

## Graphical abstract

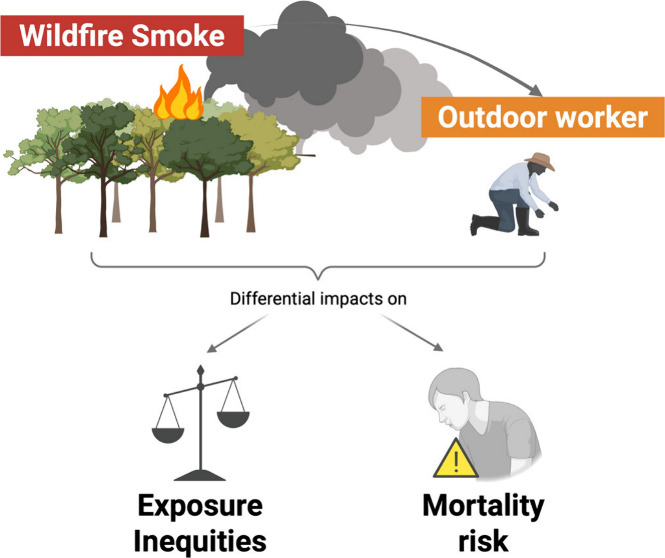

## Introduction

While the majority of the population can follow guidance to stay indoors with closed windows/doors on wildfire smoke days,^1^ the 45 million adults who regularly work outdoors in the United States (US) cannot.^2^ Outdoor workers are particularly susceptible to wildfire smoke due to direct exposures over long durations and intense exposure from increased metabolic rate, lack of ventilation, and potential coexposure with other hazards.^3^ Current policy approaches to reduce exposures rely on personal protective behaviors, as wildfire smoke can be exempted under the Clean Air Act since wildfire events are deemed “exceptional events” that US states lack the practical ability to control.^4^ The fluctuating concentrations of wildfire smoke also allow for short-term behavioral changes that can lead to reduced exposures in the general population.^5^ Research has acknowledged the potentially greater exposure and health risks for outdoor workers^6–8^ and three states have enacted regulations to protect workers,^9^ but there are no comprehensive, nationwide estimates of people exposed to wildfire smoke through outdoor occupational exposure.^10^ Our lack of understanding of workplace exposures may mask wildfire smoke-related exposure inequities and human health impacts.

Racial and ethnic groups that have been historically marginalized (hereafter, racial and ethnic minority communities) experience higher air pollution concentrations from all-source particulate matter in the US,^11^ yet studies of ambient particulate matter from wildfire smoke have observed the opposite—that some of these communities have lower concentrations.^12,13^ While previous studies have assumed that all populations within the same area experience wildfire smoke equally, racial and ethnic minority individuals are overrepresented in outdoor work such as farming, construction, and landscaping.^14^ This may lead to inequalities from workplace exposure that are not captured by comparisons of ambient concentrations alone.

Workplace exposure to wildfire smoke may also affect the health impacts experienced by individuals. We hypothesize that previously observed small and mixed existing evidence of wildfire-health risks^15^ could be partially explained by diverging behavioral patterns—i.e., people who do not work outdoors avoid wildfire smoke by remaining indoors,^5^ while people who work outdoors cannot. These disparities between outdoor concentrations and personal exposures have importance for estimating the population-level health impacts of wildfire smoke. Analyses that relate ambient concentrations to adverse health outcomes will measure a combination of the experiences of people who do and do not work outdoors, resulting in a pooled overall effect that is likely weighted toward a smaller risk due to the larger, nonoutdoor worker population in the US and their protective behaviors.

In this study, we quantified workplace exposures to wildfire smoke and explored the potential implications of differential exposure on racial and ethnic exposure inequities and mortality risk. First, we merged previously published estimates of ambient wildfire fine particulate matter (PM_2.5_) levels^16^ with employment counts and occupational characteristics (working outdoors, irregular hours) that influence wildfire smoke exposure to characterize annual work-related exposure to wildfire PM_2.5_ between 2006 and 2019 for outdoor workers among all 3,108 counties in the contiguous US. To understand the implications of this occupational exposure for racial and ethnic inequities, we then compared the racial and ethnic composition of each county with the person-days exposed to ambient and work-related wildfire smoke. Lastly, we replicated a previous analysis of ambient wildfire smoke impacts on all-cause mortality^17^ and stratified by work-related exposure to understand differential mortality effects of ambient wildfire PM_2.5_.

## Methods and Materials

This study used deidentified and publicly available data sources. Building on previous work characterizing work-related heat exposure,^18^ we characterized the number of person-days of wildfire PM_2.5_ exposure among outdoor workers in the contiguous US. We integrated previously published, county-level, daily estimates of wildfire smoke fine particulate matter (PM_2.5_) from 2006 to 2019^16^ with county-level estimates of employment counts by detailed occupational groups (760 civilian occupations, as defined by the 2010 US Bureau of Labor Statistics [BLS] Standard Occupational Classification [SOC] system, Figure S3 and Table S3).^19^ For each detailed SOC code, we considered two job characteristics that have publicly available data and that could influence exposure: (a) outdoor work and (b) irregular work hours, both derived from version 24.1 of the 2019 Occupational Information Network (Figures S4–6).^20^ Irregular work hours were incorporated to account for workers who may be exposed beyond the standard “9–5”. For each day and county between 2006 and 2019, we estimated the number of all workers who work outdoors in a given detailed SOC group within each county that are potentially exposed to smoke PM_2.5_ levels >9 μg/m^3^, a protective threshold suggested by the NIOSH in accordance with the primary annual, all-source PM_2.5_ standard set by the US Environmental Protection Agency.^3^ More details on the measures used and the approach to derive these estimates are provided in Appendix A. These estimates were aggregated spatially (e.g., nationally), occupationally (e.g., major SOC groups), and temporally (e.g., yearly and across 2006–2019) to be compared to ambient exposure levels, but our main indicator of interest for racial and ethnic inequalities and differential mortality effects was the annual, county-level rate of work-related wildfire PM_2.5_ exposure, expressed as the daily number of exposure events per 10,000 worker-days (Figure S7).

To investigate whether workplace exposures mask racial and ethnic wildfire smoke inequities, we ran two Poisson, generalized additive models to assess the same-year relationship between the racial and ethnic composition (the percent non-Hispanic White, non-Hispanic Black, or Hispanic) in each county and year with the number of person-days exposed to (i) ambient or (ii) workplace wildfire smoke. We use person-days exposed as the outcome for ambient, all-source wildfire smoke, rather than the more conventional average concentration level, to facilitate comparison between the workplace wildfire smoke metric, which is measured in person-days. We keep ambient exposure in person-days and workplace exposure in worker-days to ensure both metrics reflect individuals who have >0% probability of being exposed. Both models accounted for temporal and spatial autocorrelation, and the workplace model additionally adjusted for the population unlikely to be working, and thus unable to have work-related exposure (Appendix B).

To investigate whether wildfire smoke mortality risk differs by workplace exposure, we then fit a quasi-Poisson, panel fixed effects model using county-year data to assess the relationship of the same-year, joint exposures to ambient and work-related wildfire PM_2.5_ levels with all-cause mortality. This approach replicates the analytical design and model specifications of a previous wildfire smoke-mortality analysis using similar data (Figure S8).^17^ We obtained yearly, all-cause mortality counts for each county for all contiguous US counties between 2006 and 2019 from the National Center for Health Statistics.^21^ We assessed these relationships for the entire population, rather than the working age population, because the US workforce is increasingly aging,^22^ child labor is common, particularly in agriculture,^23^ and work-related illness from wildfire smoke could indirectly increase mortality risk for younger or older dependent family members (e.g., due to wage or health insurance loss).^24^ We included county and state-year fixed effects, natural cubic splines with 5 degrees of freedom for mean county-year temperature and precipitation,^25,26^ and an offset term of county-year total population. Details of the regression model, justification of modeling choices, and sensitivity analyses (Figure S9) are provided in Appendix C.

## Results

We found that between 2006 and 2019, there were an estimated 836 million person-days of exposure to wildfire PM_2.5_ ≥ 9 μg/m^3^ among outdoor workers in the US (Figure 1a). On average, this equated to 16 of every 10,000 US workers being exposed every day. Exposures varied substantially by day, with 233 days of the 14-year period having >1 million workers exposed. Most exposures occurred in 2007 (20% of exposures), 2011 (15%) and 2018 (12%), years where a large portion of the general US population was exposed to wildfire smoke.^16^ Farming, Fishing, and Forestry (average 76 per 10,000 workers) and Construction and Extraction (average 75 per 10,000 workers) occupations had substantially higher exposure rates than the national average (Figure 1b).

**Figure 1:**
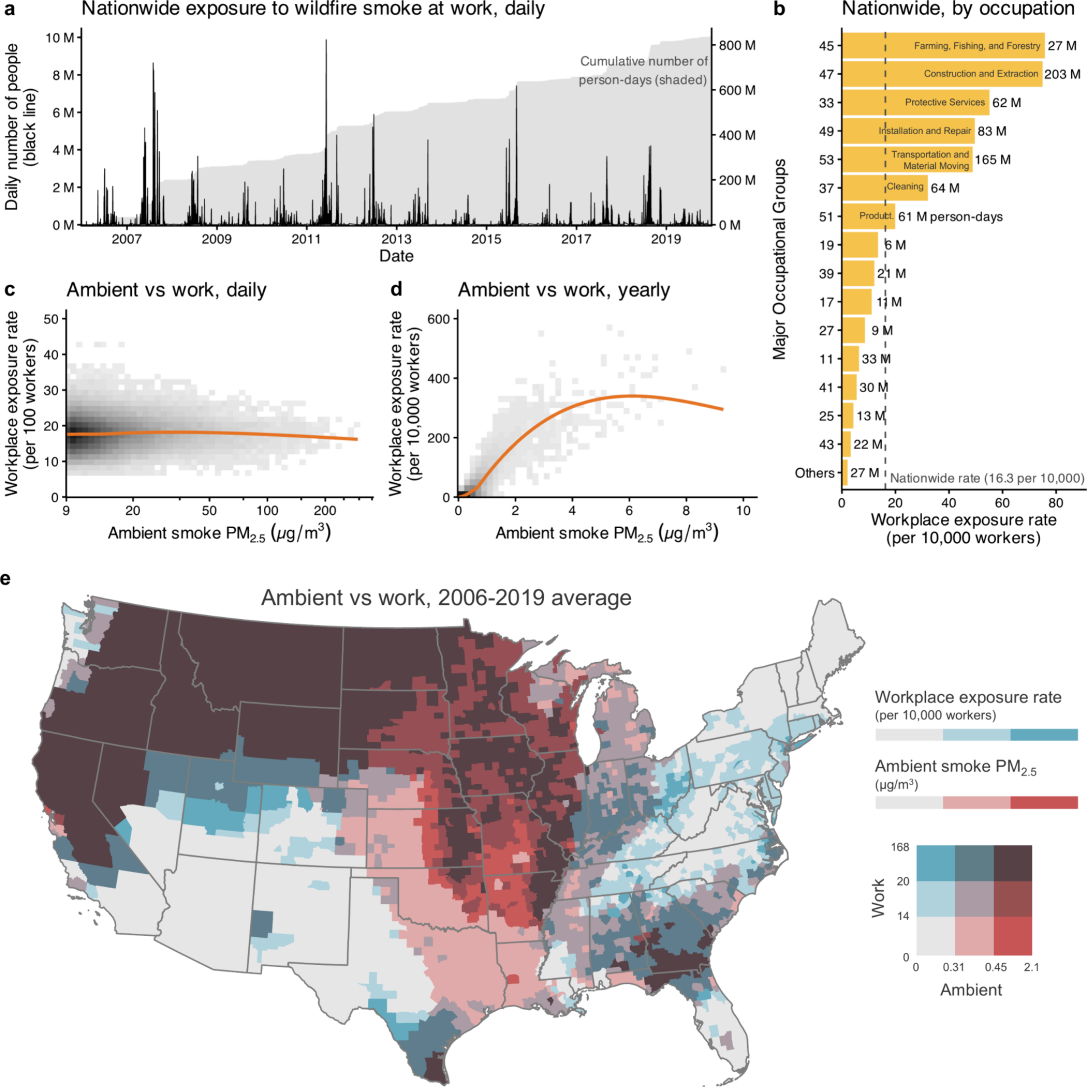
**Exposure to wildfire PM**_**2.5**_ **smoke between 2006 and 2019 among outdoor workers in the contiguous US and comparison to ambient levels. (a)** Daily number of people exposed (black line) and cumulative number of person-days exposed (gray shaded area). **(b)** Bar chart of the workplace exposure rate of wildfire smoke by major occupational group during 2006–2019 (bar labels present total number of person-days exposed). **(c)** The relationship between daily ambient smoke PM_2.5_ (μg/m^3^) vs the daily rate of work-related exposure per 100 workers on county-days ≥ 9 μg/m^3^ during 2006–2019. Shaded area reflects density of observations, with darker shades indicating more observations. Ambient smoke levels are log-transformed to make lower values more visible, since there are few days >50 μg/m^3^. **(d)** The relationship between annual average, ambient smoke PM_2.5_ (μg/m^3^) vs the annual rate of work-related exposure per 10,000 workers for every county-year during 2006–2019. Shaded area reflects density of observations, with darker shades indicating more observations. **(e)** Bivariate map of the daily rate of work-related exposure per 10,000 workers and average ambient smoke PM_2.5_ (μg/m^3^) at the county-level during 2006–2019. Note: major standard occupational classification (SOC) group titles: 11 – Management Occupations; 17 – Architecture and Engineering Occupations; 19 – Life, Physical, and Social Science Occupations; 25 – Educational Instruction and Library Occupations; 27 – Arts, Design, Entertainment, Sports, and Media Occupations; 33 – Protective Service Occupations; 37 – Building and Grounds Cleaning and Maintenance Occupations; 39 – Personal Care and Service Occupations; 41 – Sales and Related Occupations; 43 – Office and Administrative Support Occupations; 45 – Farming, Fishing, and Forestry Occupations; 47 – Construction and Extraction Occupations; 49 – Installation, Maintenance, and Repair Occupations; 51 – Production Occupations; 53 – Transportation and Material Moving Occupations.

Daily ambient wildfire PM_2.5_ smoke concentrations correlated poorly with work-related exposures (Figure 1c). While annual average ambient wildfire PM_2.5_ smoke concentrations correlated more strongly with work-related exposure, increasing ambient levels at the tails (<0.5 or >4 μg/m^3^) were not strongly correlated with higher work-related exposure (Figure 1d). When averaging across 2006–2019, Figure 1e illustrates that there are many counties with the highest ambient wildfire PM_2.5_ but the lowest workplace exposure rates. This suggests that characterizing workplace exposures can provide information about wildfire smoke exposure beyond the information ambient levels provide.

Ambient measures also masked racial and ethnic exposure inequities to wildfire PM_2.5_ smoke (Figure 2A). Compared to counties with 0% non-Hispanic White individuals, counties with higher proportions of non-Hispanic White individuals in the population were more exposed to ambient wildfire PM_2.5_, on average. Meanwhile, counties with higher proportions of non-Hispanic White individuals were less exposed to wildfire PM_2.5_ at work compared to counties with 0% of these individuals. Conversely, while counties with higher proportions of non-Hispanic Black and Hispanic individuals were less exposed to ambient wildfire PM_2.5_, both were more exposed at work, particularly counties with higher proportions of Hispanic individuals.

**Figure 2:**
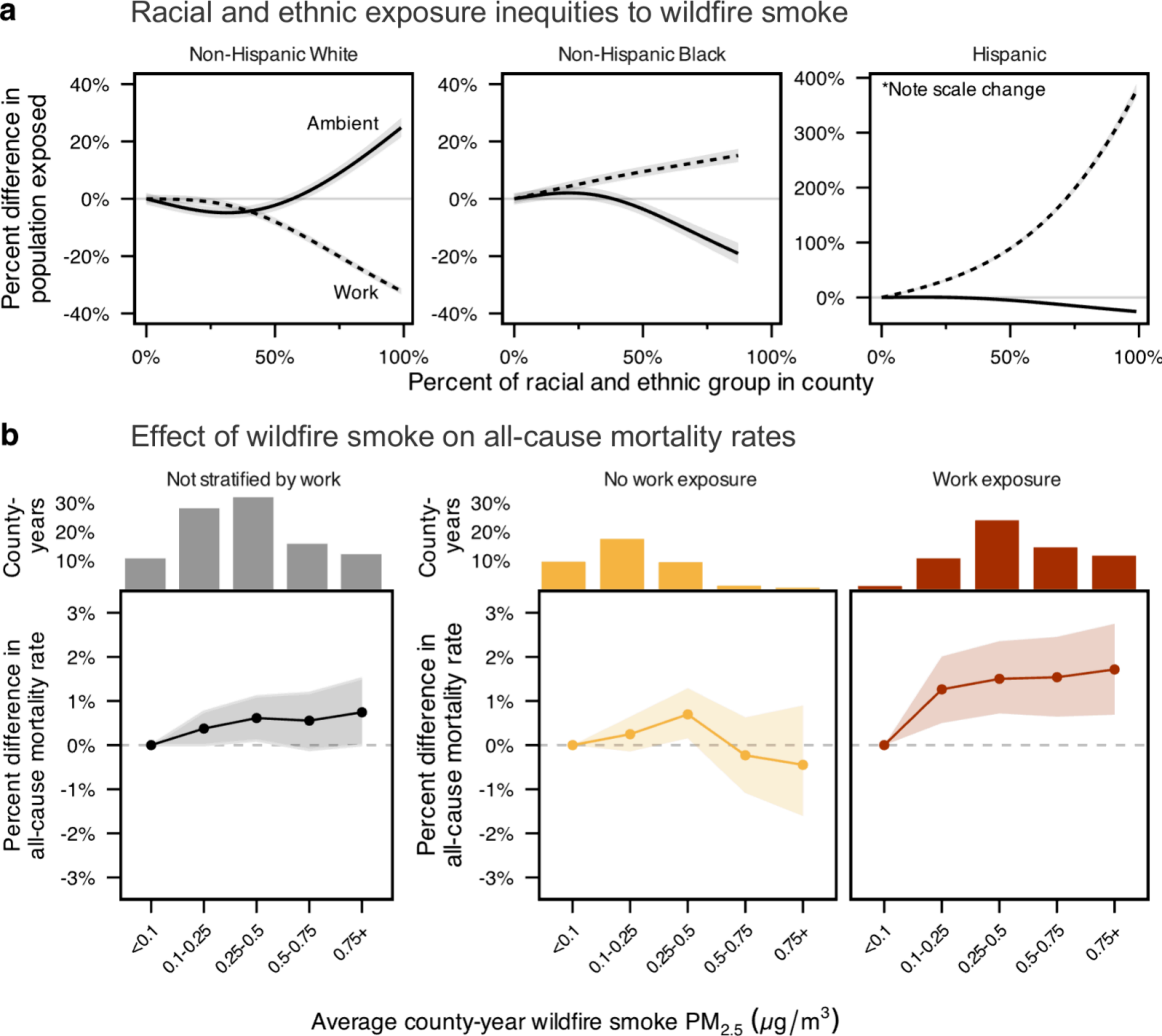
**Workplace exposure to wildfire smoke PM**_**2.5**_ **may mask wildfire-related exposure inequities and all-cause mortality risk. (a)** County-year relationship between the racial and ethnic composition of a county and the rate of ambient (solid line) and workplace (dashed line) wildfire PM_2.5_ exposure during 2006–2019. Percent differences are calculated relative to counties with 0% of the population identifying as the respective racial and ethnic group. **(b)** County-year association from panel fixed effect quasi-Poisson regression models between average, ambient smoke PM_2.5_ and all-cause mortality rates between 2006 and 2019 among all counties (left panel) and among counties with no (0 exposure-days per 10,000 workers) or any (>0 exposure-days per 10,000 workers) workplace exposure (right two panels).

Differential workplace exposure was associated with detectable differences in all-cause mortality rates (Figure 2b**;** Table S2). Consistent with previous studies,^17,27^ we found an increasing trend between same-year annual ambient wildfire smoke PM_2.5_ and all-cause mortality rates when all counties were pooled together, although the effects at higher averages >0.5 μg/m^3^ were not statistically significant. Among county-years with no workplace exposure (Figure S1), mortality risk peaked at 0.25–0.5 μg/m^3^ (0.7% higher mortality rate, 95% CI: 0.2 to 1.2%) relative to no/minimal ambient smoke PM_2.5_ (<0.1 μg/m^3^), with no increase in mortality risk for ambient annual concentrations >0.5 μg/m^3^. In contrast, county-years with workplace exposure to wildfire smoke (Figure S2) displayed statistically significantly higher all-cause mortality rates (1.2 to 1.7%) across all smoke PM_2.5_ concentrations relative to no/little ambient PM_2.5_, consistent with our hypothesis that outdoor workers cannot avoid exposure. These observations are qualitatively similar even when examining all-cause mortality rates among the working-age population (15–64 years old) rather than all ages (Figure S10).

## Discussion

Collectively, these findings suggest that work-related wildfire smoke exposures may play an important role in masking wildfire smoke exposure inequities and all-cause mortality. The observed inequities indicate that racial and ethnic minority communities, particularly Hispanic, may disproportionately bear the health burden of wildfire smoke, although further research is needed to investigate potential health disparities. The differential mortality associations suggest that wildfire smoke adaptation measures may be beneficial in areas with higher workplace exposure to wildfire smoke.

There are several factors to consider when interpreting our results. Our findings are limited to the United States, which may be generalizable to other developed nations (e.g., Canada, Australia) but may understate the influence of workplace exposures in developing nations without a robust occupational regulatory system. We may have mischaracterized work-related exposure in some counties, although we made specific analytic decisions that avoid strong bias in either direction (see Appendix A). Our approach cannot estimate wildfire smoke exposure by detailed job types. This analysis does not consider other individual characteristics, like income or housing quality, which affect individual exposures. Future studies should consider incorporating individual-level information. While our regression approach replicates previous, plausibly causal estimations of wildfire smoke and mortality,^17^ there may be additional, time-varying, residual confounding that could bias our observed effects (e.g., short-term labor “shocks”, baseline health status). However, these confounders would need to be correlated with wildfire smoke PM_2.5_ within counties. Our observed differential mortality effect is sensitive to the threshold that defines work-related exposure (9 μg/m^3^); however, this threshold was guided by available recommendations.^3^ This makes it difficult to disentangle the contribution of workplace exposure or the higher exposure levels more generally to the observed differential effect. Our approach is also limited by the modifiable areal unit problem; conducting the analysis using an arbitrary administrative boundary (i.e., county) to assess spatial patterns in workplace exposure could result in different exposure patterns at a finer spatial scale. However, since much of the United States is impacted by long-range transport of wildfire smoke, we suspect that finer-scale estimates are relatively homogeneous across a county and that the exposure-mortality relationships would remain consistent with our findings.

Regardless of these limitations, the differential mortality associations are plausible for several reasons. Negative effects of smoke on emergency department visits at high daily concentrations have been observed previously,^28^ suggesting those without workplace exposure, such as office workers, could be altering their behaviors to avoid wildfire smoke due to greater job autonomy than that of outdoor workers.^5,15^ Meanwhile, outdoor workers have limited options to avoid and control exposure to wildfire smoke and are often exposed to wildfire smoke for prolonged periods of time (>8 h/day). The few controls that exist for outdoor workers, such as placing workers in semipermanent structures, implementing air filtration systems, rotating work schedules, altering work intensity, and providing respirators,^3^ were unlikely to have been implemented in many workplaces as of 2019 due to a lack of workplace regulations. Only three states (California, Oregon, and Washington) have since implemented regulations, despite the increased geographic and temporal spread of wildfire smoke in the 2020s.^29^ The lack of sufficiently protective regulations might be burdening racial and ethnic minority communities the most, who we observed to experience disproportionately higher work-related exposures but lower ambient exposures. This supports previous hypotheses that occupational constraints may result in larger wildfire smoke disparities.^12^ Together, our findings highlight the need to protect outdoor workers, particularly those who identify as a racial and ethnic minority, and consider occupational health in wildfire smoke adaptation measures to reduce the population health burden of wildfire smoke.

## Supplementary Material



## Data Availability

Yearly county breakdowns of workplace wildfire smoke exposure rates are available at https://doi.org/10.5281/zenodo.20435507. For daily exposure rate estimates, contact Abas Shkembi (email: ashkembi@umich.edu). All data underlying the analysis are publicly available. Major employment counts and sociodemographic compositions are available from the `tidycensus̀ R package (https://cran.rproject.org/web/packages/tidycensus/index.html). Daily, ambient wildfire smoke levels were previously published by Childs et al. (https://github.com/echolab-stanford/daily-10km-smokePM). Metropolitan/nonmetropolitan-level detailed employment counts are available by the Bureau of Labor Statistics Occupational Employment and Wage Statistics (https://www.bls.gov/oes/tables.htm). Workplace characteristics are made publicly available from version 24.1 of the Occupational Information Network (https://www.onetcenter.org/db_releases.html). Mortality data are made publicly available by the CDC National Center for Health Statistics on the WONDER platform (https://wonder.cdc.gov/deaths-by-underlying-cause.html). Replication code required to reproduce the results are maintained in the following GitHub repository: https://github.com/abasshkembi/workplace-wildfire-smoke.

## Notes

List of Supplementary Tables - Table S1 - NIOSH Exposure Control Categories - Table S2 - documentation of results shown in Figure 2b - Table S3 - steps to construct work-related wildfire smoke exposure rates. List of Supplementary Figures - Figure S1 - ambient wildfire smoke among counties with no work-related exposure - Figure S2 - ambient wildfire smoke among counties with any work-related exposure - Figure S3 - flowchart depicting construction of work-related exposure rate - Figure S4 - histogram of outdoor work by detailed occupation - Figure S5 - outdoor workers by county - Figure S6 - irregular working hours by detailed occupation - Figure S7 - yearly rate of work-related wildfire smoke exposure by county - Figure S8 - sensitivity analysis of wildfire smoke-mortality analysis - Figure S9 - sensitivity analysis of handling suppressed mortality counts - Figure S10 - sensitivity analysis of using working-age population instead of all ages. Supplemental Methods and Results - Appendix A. Characterizing workplace exposure to wildfire PM_2.5_ - Appendix B. Relationship between racial and ethnic composition and ambient and work-related exposure to wildfire PM_2.5_ - Appendix C. Relationship between ambient wildfire PM_2.5_, workplace exposure, and mortality relationship (PDF)
